# No clear associations of adult BMI and diabetes mellitus with non-muscle invasive bladder cancer recurrence and progression

**DOI:** 10.1371/journal.pone.0229384

**Published:** 2020-03-25

**Authors:** Jelle Evers, Anne J. Grotenhuis, Katja K. H. Aben, Lambertus A. L. M. Kiemeney, Alina Vrieling

**Affiliations:** 1 Department for Health Evidence, Radboud Institute for Health Sciences, Radboud University Medical Center, Nijmegen, The Netherlands; 2 Netherlands Comprehensive Cancer Organisation, Utrecht, The Netherlands; 3 Department of Urology, Radboud Institute for Health Sciences, Radboud University Medical Center, Nijmegen, The Netherlands; Johns Hopkins University School of Medicine, UNITED STATES

## Abstract

**Background:**

Non-muscle invasive bladder cancer patients are at high risk for tumour recurrence and progression, hence an intensive follow-up procedure is recommended which is costly. Identification of factors that are associated with the risk of recurrence and progression may enable personalized follow-up schedules. Obesity and diabetes mellitus may be associated with a worse prognosis, but the evidence is limited and inconsistent. Our objective was to determine the associations of BMI and diabetes mellitus with risks of recurrence and progression among non-muscle invasive bladder cancer patients.

**Methods:**

A population-based cohort of patients diagnosed with non-muscle invasive bladder cancer between 1995 and 2010 was retrospectively identified from the Netherlands Cancer Registry and invited to participate in the Nijmegen Bladder Cancer Study (n = 1,433). Average weight during adult life, height, and diabetes mellitus diagnosis were self-reported by use of a questionnaire. Clinical follow-up data were retrieved from medical files. Associations were quantified using proportional hazard analyses. For all analyses, minimal adjustment sets were selected using a Directed Acyclic Graph.

**Results:**

Fourteen percent of the patients indicated to be diagnosed with diabetes mellitus, and more than half was overweight (45%) or obese (9%). Compared to healthy weight, overweight and obesity were not associated with risk of recurrence (adjusted hazard ratio (HR) = 1.02; 95% confidence interval (CI): 0.86–1.22, and HR = 1.02; 95% CI: 0.76–1.38, respectively) and overall progression (HR = 1.04; 95% CI: 0.74–1.44, and HR = 1.20; 95% CI: 0.69–2.09, respectively). Also, no clear associations of diabetes mellitus with risk of recurrence (HR = 1.22; 95% CI: 0.98–1.54) and overall progression (HR = 1.16; 95% CI: 0.76–1.76) were found.

**Conclusion:**

Average BMI during adult life and diabetes mellitus were not clearly associated with risk of recurrence or progression in non-muscle invasive bladder cancer. Prospective cohort studies with detailed information on BMI and diabetes mellitus before and after diagnosis are needed to confirm these findings.

## Introduction

With 550,000 new cases each year, urinary bladder cancer (UBC) is the tenth most frequently diagnosed cancer worldwide, particularly occurring in high income countries [1]. Patients with muscle invasive bladder cancer (MIBC) have a low five-year survival rate of up to approximately 35% [2, 3]. Patients with non-muscle invasive bladder cancer (NMIBC) have a high five-year disease-specific survival, ranging between 88.7% and 98.5%. However, the five-year risk of recurrence is 28.3% to 51.7% and the five-year risk of progression is 4.6% to 19.8% [4]. Therefore, regular check-ups after initial treatment are recommended and resection of recurrent tumours and subsequent treatment are frequently required [5]. As a consequence, UBC has the highest lifetime treatment costs per patient of all cancers, which significantly impacts the total healthcare costs of oncology [6]. To predict prognostic outcomes in NMIBC patients, the European Organization for Research and Treatment of Cancer (EORTC) and Spanish Urology Association for Oncological Treatment (CUETO) designed scoring tables [7, 8]. These tables focus mainly on primary tumour characteristics. However, lifestyle factors such as smoking and obesity, as well as diabetes mellitus (DM), may also be relevant in the prognosis of NMIBC [9], and may contribute to more personalized follow-up schedules.

Obesity and DM have an estimated global prevalence above 650 and 400 million cases, respectively [10, 11]. More than 90% of all DM diagnoses in high income countries concern type 2 diabetes mellitus (DM2) which is strongly related to overweight [11]. Both obesity and DM2 are associated with increased risks of a variety of cancers, including UBC [12–15]. A meta-analysis of Sun et al. (2015) reported a relative risk (RR) of 1.10 (95% confidence interval (CI): 1.06–1.14) for UBC risk in obese compared with healthy weight people [16]. For diabetics compared with non-diabetics, a meta-analysis of Zhu et al. (2013) showed a RR of 1.35 (95% CI: 1.17–1.56) for UBC risk [17].

Proposed mechanisms for the effect of obesity and DM2 on cancer include the occurrence of insulin resistance and consequently hyperinsulinemia [13, 18]. This has a direct effect by increased binding of insulin to the tumour cell’s insulin-receptor and an indirect effect by increasing the concentration of free insulin-like-growth-factor-1 (IGF-1), resulting in increased tumour growth. Moreover, adipose tissue is characterised by macrophage infiltration, which plays an important role in inflammation. Macrophages and adipocytes produce pro-inflammatory factors, resulting in elevated concentrations of circulating tumour necrosis factor-α (TNF-α), interleukin-6 (IL-6) and C-reactive protein (CRP), which are beneficial for tumour growth [13, 18]. Mechanisms may differ by cancer type, and the exact mechanisms for UBC risk, recurrence and progression remain to be elucidated.

Although obesity and DM seem to be associated with UBC risk, only few studies are published on their associations with risk of tumour recurrence or progression in NMIBC patients. In a recent meta-analysis based on three historical cohort studies, our group found a pooled hazard ratio (HR) of 1.82 (95% CI: 1.12–2.95) for recurrence and 1.90 (95% CI: 0.93–3.88) for progression in obese versus healthy weight NMIBC patients [9, 19–21]. Hwang et al. (2011) found increased risks of recurrence and progression for diabetic compared with non-diabetic NMIBC patients [22]. Two studies comparing diabetic NMIBC patients with or without metformin use, a first line glucose-lowering drug, with non-diabetic NMIBC patients showed contradictory results [23, 24].

Thus, evidence for body mass index (BMI) and DM in NMIBC prognosis is limited and inconsistent, based on relatively small cohort studies, and restricted to BMI at diagnosis which may have been affected by the disease. Since such evidence may contribute to personalized follow-up schedules, we aimed to determine the associations of average BMI during adult life and DM with risk of recurrence and progression in a large population-based cohort of NMIBC patients.

## Methods

### Study population and data collection

**Fig 1:**
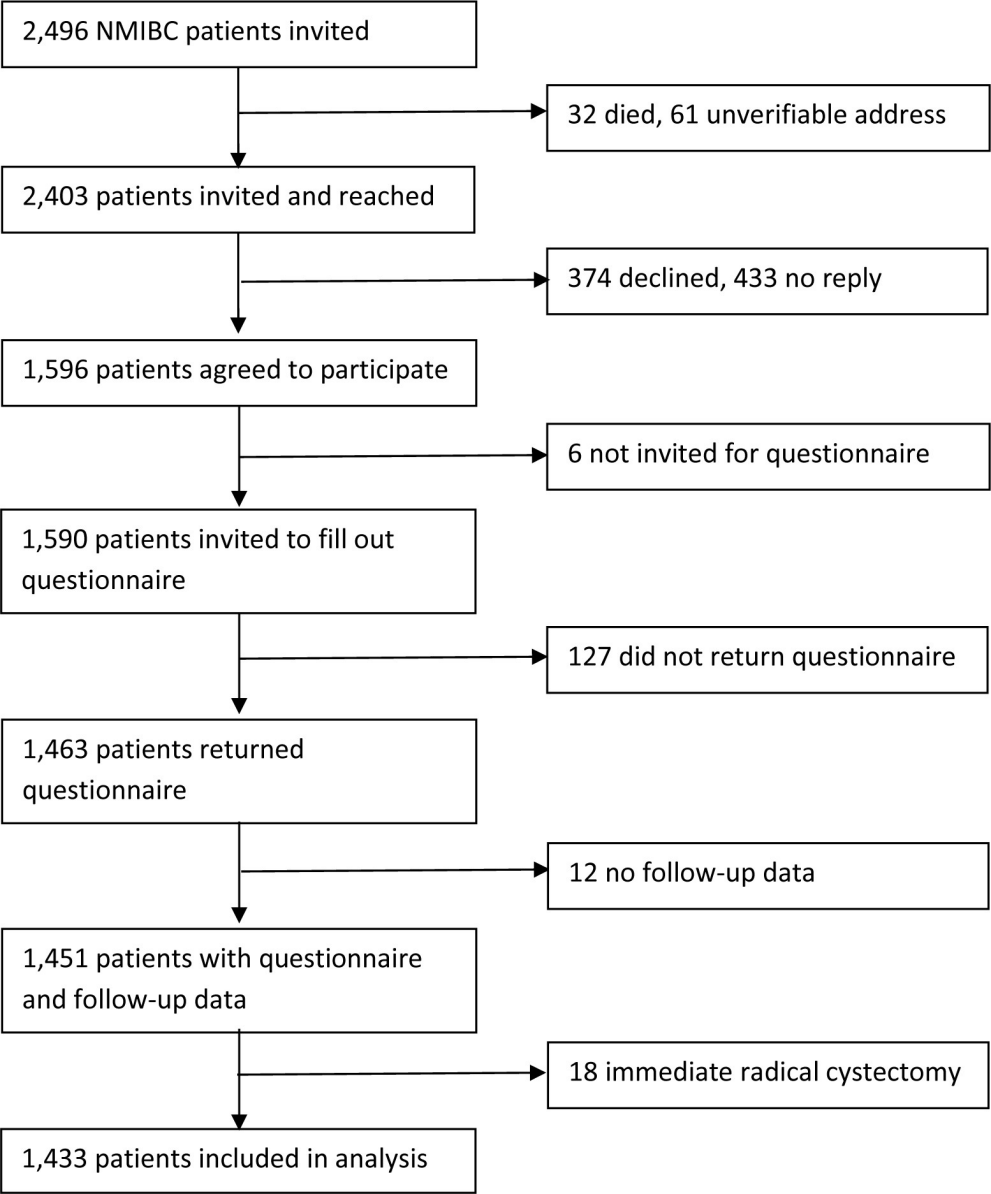
Flow diagram.

NMIBC patients were selected from the Nijmegen Bladder Cancer Study (NBCS) [25]. NMIBC patients who are registered in the Netherlands Cancer Registry (NCR), held by the Netherlands Comprehensive Cancer Organisation, were selected and invited to participate in the NBCS in four batches. Invitations for the first, second, third, and fourth batch were sent in May 2007, January 2009, November 2010, and February 2012, respectively. Only NMIBC patients under the age of 75 years and who were diagnosed between 1995 and 2010 in one of seven hospitals in the mid-eastern part of the Netherlands were invited. Also, a non-overlapping series of 280 NMIBC patients who were previously recruited for a separate study on gene-environment interactions was included [25]. Of all invited patients, 59% responded and filled out a questionnaire on general characteristics, medical history, lifestyle, and family history of cancer. The median time between diagnosis and filling out the questionnaire was 2.8 years (interquartile range: 1.8–6.7 years). Data on the primary tumour, treatment, and clinical follow-up were retrieved from medical files. Since clinical follow-up was assessed in the framework of a previous genome wide association study (GWAS), follow-up data were only available for NMIBC patients who donated a blood sample for DNA isolation [26]. Further, only patients who returned a questionnaire and did not undergo radical cystectomy directly after being diagnosed with the primary NMIBC were included (Fig 1).

The NBCS was approved by the Institutional Review Board of the Radboud university medical center. All participants gave written informed consent.

### Exposure definitions

Patients were asked to report their average body weight during adult life and their height. Body weight was divided by the square of height to calculate average BMI during adult life, which will be further referred to as (adult) BMI. BMI was used as continuous and categorical variable. Obesity was defined as BMI ≥30.0, overweight as BMI ≥25.0 and <30.0, healthy weight as BMI ≥18.5 and <25.0, and underweight as BMI <18.5 kg/m^2^. No obesity was defined as a BMI <30.0 kg/m^2^ [27]. Only 2 patients had underweight, and were excluded from the analyses on BMI.

Patients were asked whether they were ever diagnosed with DM by a physician and at which age. Patients with missing values for DM diagnosis were assumed not having DM. Since DM type was not assessed, we classified type by using a proxy of age at DM diagnosis and BMI. Type 1 diabetes mellitus (DM1) was defined as a diagnosis at age ≤30 years, or at age 31–40 years in combination with having no obesity. Type 2 diabetes mellitus (DM2) was defined as a diagnosis at age >40 years, or at age 31–40 years in combination with being obese.

### Lifestyle factors

Patients were asked for their smoking status at recruitment, age at smoking initiation and cessation, number of cigarettes smoked per day and duration of smoking in years. Smoking status at diagnosis and cumulative smoking exposure (in pack-years) was calculated using these variables, as previously described in more detail [28].

We also asked patients how many hours per week they spent walking, cycling, and sporting during adult life until 2 years before the NMIBC diagnosis, and with which frequency: <1 time/week, 1–2 times/week, 3–5 times/week or >5 times/week. For those with missing values for duration but known frequencies, missing values were imputed with calculated average durations per frequency stratum. The weekly duration of walking, cycling and sporting were summed to calculate the weekly duration of total physical activity.

### UBC family history

Patients were asked to report whether a parent, sibling, or child ever had cancer, and if so, the type and year of diagnosis. Those who did not report a first-degree relative with UBC were included as having no family history.

### Clinical data

Detailed clinical data concerning age at diagnosis, tumour stage, tumour grade, tumour number (single or multiple), tumour size (<3cm and ≥3cm), presence of concomitant carcinoma in situ (CIS), histological type, and initial treatment were collected through a medical file survey [28]. Tumour stage and grade were recorded according to the final conclusion in the pathology report. Tumours with World Health Organisation (WHO) 1973 differentiation grade 1 or 2, WHO/International Society of Urological Pathology (ISUP) 2004 low grade, or Malmström (Modified Bergkvist) grade 1 or 2a were considered low-grade tumours. We classified tumours with WHO 1973 differentiation grade 3, WHO/ISUP 2004 high grade, or Malmström (Modified Bergkvist) grade 2b or 3 as high-grade [29, 30]. Tumour aggressiveness was classified according to the risk of progression as follows: low-grade Ta tumours were classified as low-risk while all stage T1 tumours and CIS were classified as high-risk [31].

### Outcome definitions

Outcomes were assessed by one of the authors (AJG) up to 5 years after diagnosis. Predefined instructions and a codebook were used to obtain consistency in retrieving the data. About 5% of medical records were seen twice to check consistency. A recurrence was defined as a new histologically confirmed tumour in the urinary bladder or prostatic urethra, after at least one tumour-negative follow-up cystoscopy or a radical re-transurethral resection of the primary tumour. For progression, the consensus definition of the International Bladder Cancer Group was used: the first occurrence of stage or grade progression, local or distant metastasis, and cystectomy for therapy-resistant disease, hereafter referred to as overall progression [32]. Additionally, a stricter definition was used where progression was defined as transition to MIBC (stage ≥T2).

Recurrence-free survival (RFS) and progression-free survival (PFS) were defined as the time between the date of diagnosis of the primary NMIBC and the date of first recurrence or recurrence with progression, respectively. In patients without recurrence or progression, follow-up was censored at the date of the last urological check-up, the date of death, or 5-year follow-up, whichever came first in time.

### Statistical analyses

Standard descriptive statistics were calculated to describe patient and tumour characteristics for the total cohort, and by BMI and DM categories. Risks of recurrence and progression were calculated using the Kaplan-Meier method and presented in cumulative risk plots, stratified by exposure groups. Differences between the exposure groups were investigated using log rank tests. To calculate HRs and corresponding 95% CIs for the associations of BMI and DM with UBC outcomes, univariable and multivariable Cox proportional hazard analyses were performed for both RFS and PFS. The proportional hazards assumption was tested by modelling time-dependent covariables and using Schoenfeld residuals [33]. The assumption was fulfilled for all exposures of interest and potential covariables, except for BMI in relation to overall progression.

Sets of covariables for sufficient adjustment were initially selected using a Directed Acyclic Graph (DAG). This method has the advantage of keeping the included number of covariables to a minimum, resulting in more precise estimations of effects. The DAG was created using the software DAGitty [34]. First, the exposure, outcomes, covariables, and their causalities and associations known from literature were graphically depicted. The implications of independence between variables in the DAG were then tested in our data, using the software R, version 3.2.5 with the packages DAGitty [35], foreign 0.8–66 [36] and ggm 2.3 [37]. When this evaluation showed dependence between variables that were initially assumed independent, the DAG was improved by linking the variables. We refer to S1 Fig for the final DAG. Minimal sets of covariables for sufficient adjustment were subsequently selected from the final DAG, also using the software DAGitty. Covariables were selected for adjustment so that confounding pathways present in the DAG were blocked. It should be noted that adjusting for confounders deemed important (e.g. smoking or initial treatment) may not be required as a consequence of adjusting for other covariables in the same pathway. Details on the technical background of selecting covariables as applied by the software are described elsewhere [38].

In addition to the covariables that were selected using the DAG, most primary tumour characteristics of the EORTC and CUETO risk prediction tables were added to the adjustment sets [7, 8]. Prior recurrence rate was not included since all patients were newly diagnosed with NMIBC. Tumour size and number of tumours were not reported in the medical files of most patients, and therefore not included. The final adjustment set for the associations of BMI with recurrence and progression consisted of age at time of diagnosis, gender, highest completed level of education, weekly duration of physical activity, history of UBC among first degree relatives, tumour stage, tumour grade and presence of concomitant CIS. The final adjustment set for the associations of DM with recurrence and progression consisted of age at time of diagnosis, gender, BMI classes, tumour stage, tumour grade and presence of concomitant CIS. Patients with missing data on covariables (~3%) were excluded from the adjusted analyses.

Analyses were performed using the software IBM SPSS Statistics for Windows, release 22.0.0.1 (IBM Corp., Armonk, NY, USA), except for the analyses of testing the DAG. P-values ≤0.05 were considered statistically significant.

## Results

### Population characteristics

We included 1433 NMIBC patients. Patient characteristics on lifestyle and medical history, and characteristics regarding their primary tumour and the initially received treatment are presented in Table 1. The median age at time of NMIBC diagnosis was 64 years (interquartile range: 56–70 years). Most patients were male (83%) and current (37%) or former cigarette smokers (43%). The median adult BMI was 25.3 kg/m^2^ (interquartile range: 23.7–27.2 kg/m^2^), and 14% of the patients indicated to have been diagnosed with DM. Almost all patients had a urothelial cell carcinoma (99.4%) and received transurethral resection of the bladder tumour, with (50%) or without (47%) adjuvant intravesical instillations. The majority of patients had a Ta (70%) and low grade tumour (64%) and were at low risk of progression (59%). Most characteristics did not differ by BMI classes and DM status, except for educational level and physical activity which were lower among overweight and obese compared to healthy weight patients and patients with DM compared to no DM (S1 and S2 Tables).

**Table 1:** Patient characteristics on lifestyle and medical history, and characteristics regarding their primary bladder tumour and initial treatment.

		Patients (N = 1433)	
		n	(%)
**Demographic characteristics**			
Age in years, median (P_25_, P_75_) ^a)^		64.0	(56.0, 70.0)
Male		1187	(82.8)
Ethnicity ^b)^			
	Dutch	1294	(90.3)
	Non-Dutch	139	(9.7)
Highest completed level of education ^c)^			
	Low	794	(55.4)
	Intermediate	308	(21.5)
	High	329	(23.0)
	Unknown	2	(0.1)
**Lifestyle factors**			
Adult BMI in kg/m^2^			
	<18.5	2	(0.1)
	≥18.5 and <25.0	644	(44.9)
	≥25.0 and <30.0	642	(44.8)
	≥30.0	128	(8.9)
	Unknown	17	(1.2)
	Median (P_25_, P_75_)	25.3	(23.7, 27.2)
Cigarette smoking status ^a)^			
	Current	531	(37.1)
	Former	620	(43.3)
	Never	259	(18.1)
	Unknown	23	(1.6)
Cigarette pack-years, median (P_25_, P_75_) ^a)^ ^d)^		22.0	(12.0, 36.0)
Weekly duration of physical activity in hours, median (P_25_, P_75_) ^e)^		9.0	(5.5, 15.0)
**Medical history**			
Diagnosed with diabetes mellitus ^f)^			
	Yes	198	(13.8)
	No ^g)^	1235	(86.2)
Diabetes mellitus type ^f)^ ^h)^			
	Type 1	13	(6.6)
	Type 2	124	(62.6)
	Unknown	61	(30.8)
UBC history among first degree relatives ^f)^			
	Yes	98	(6.8)
	No	1335	(93.2)
**Primary tumour characteristics**			
Stage, TNM 2002 classification			
	Ta	1008	(70.3)
	CIS	54	(3.8)
	T1	349	(24.4)
	Unknown	22	(1.5)
Grade ^i)^			
	Low grade (G1 or G2)	921	(64.3)
	High grade (G3)	496	(34.6)
	Unknown	16	(1.1)
Risk of progression ^j)^			
	Low	840	(58.6)
	High	573	(40.0)
	Unknown	20	(1.4)
Histology			
	Urothelial cell carcinoma	1424	(99.4)
	Other, including combinations of histology types	2	(0.1)
	Unknown	7	(0.5)
Initial treatment			
	TURT with one intravesical chemotherapy instillation	668	(46.6)
	Adjuvant intravesical chemotherapy	443	(30.9)
	Adjuvant intravesical immunotherapy	261	(18.2)
	Adjuvant intravesical chemo- and immunotherapy	18	(1.3)
	Other	1	(0.1)
	Unknown	42	(2.9)
Concomitant CIS			
	Yes	105	(7.3)
	No	1310	(91.4)
	Unknown	18	(1.3)
Focality			
	Unifocal	786	(54.8)
	Multifocal	564	(39.4)
	Unknown	83	(5.8)
Size in cm			
	<3	193	(13.5)
	≥3	111	(7.8)
	Unknown	1129	(78.8)

P_25_: 25^th^ percentile; P_75_: 75^th^ percentile; BMI: body mass index; CIS: carcinoma in situ; TURT: transurethral resection of the bladder tumour.

[a] At the time of non-muscle invasive bladder cancer diagnosis.

[b] Based on the country of birth of the patients and their parents.

[c] Based on the International Standard Classification of Education: low level includes elementary, lower vocational and intermediate general education; intermediate level includes intermediate vocational and higher general education; high level includes higher vocational education and university.

[d] Presented for current and former cigarette smokers; unknown for 32 (former) smokers (2.8%).

[e] Based on the weekly duration of walking, cycling, and sporting during adult life until 2 years before diagnosis; unknown for 25 patients (1.7%).

[f] At the time of filling out the questionnaire.

[g] 66 patients (4.6%) had a missing value for diagnosis of diabetes mellitus and were included as not diagnosed.

[h] Presented for patients diagnosed with diabetes mellitus; type 1 was defined as diabetes mellitus diagnosis at age ≤30 years, or at age 31–40 years in combination with being not obese; type 2 was defined as diabetes mellitus diagnosis at age >40 years, or at age 31–40 years in combination with being obese.

[i] Tumours with WHO 1973 differentiation grade 1 or 2, WHO/ISUP 2004 low grade, or Malmström (Modified Bergkvist) grade 1 or 2a were considered low-grade tumours. Tumours with WHO 1973 differentiation grade 3, WHO/ISUP 2004 high grade, or Malmström (Modified Bergkvist) grade 2b or 3 as high-grade.

[j] Based on the European Association of Urology guidelines: low risk includes Ta-stage with low grade, high risk includes Ta-stage with high grade, T1-stage, T2-stage, T3-stage, T4-stage, and CIS.

### Associations of adult BMI and DM with recurrence

In the first 5 years after diagnosis, 606 patients experienced at least one recurrence. The Kaplan-Meier method yielded a five-year risk of 48% (95% CI: 45%-50%) for recurrence.

BMI was not associated with risk of recurrence (S2 Fig and Table 2). The adjusted HRs for overweight and obesity, with healthy weight as reference, were 1.02 (95% CI: 0.86–1.22) and 1.02 (95% CI: 0.76–1.38), respectively. The analysis for continuous BMI yielded an adjusted HR of 1.00 (95% CI: 0.98–1.02) per 1 kg/m^2^ increase in BMI. Overall DM was associated with a non-statistically significant increased risk of recurrence (HR: 1.22, 95% CI: 0.98–1.54), compared with no DM. DM1 and DM2 were not statistically significantly associated with risk of recurrence compared with no DM (HR: 1.39, 95% CI: 0.66–2.95 and HR: 1.00, 95% CI: 0.75–1.33, respectively).

**Table 2:** Crude and adjusted hazard ratios (HR) with corresponding 95% confidence intervals (CI) for the associations of adult BMI and diabetes mellitus with risk of recurrence ^a)^ among non-muscle invasive bladder cancer patients.

		*Crude analyses*				*Adjusted analyses* ^b)^			
		Number at risk	Events ^c)^	HR	(95% CI)	Number at risk	Events ^c)^	HR	(95% CI)
BMI classes in kg/m^2^ ^d^)									
	≥18.5 and <25.0	644	273	Reference		623	264	Reference	
	≥25.0 and <30.0	642	273	1.00	(0.84–1.18)	621	263	1.02	(0.86–1.22)
	≥30.0	128	53	0.96	(0.72–1.29)	125	53	1.02	(0.76–1.38)
Continuous BMI in kg/m^2^ ^d)^		1414	599	1.00	(0.98–1.02)	1369	580	1.00	(0.98–1.02)
Obesity ^d)^									
	No	1286	546	Reference		1244	527	Reference	
	Yes	128	53	0.96	(0.73–1.28)	125	53	1.01	(0.76–1.34)
Diabetes mellitus status ^e)^									
	No	1235	511	Reference		1201	496	Reference	
	Yes	198	95	1.18	(0.95–1.46)	191	94	1.22	(0.98–1.54)
Diabetes mellitus type ^e)^									
	No diabetes mellitus	1235	511	Reference		1201	496	Reference	
	Type 1	13	7	1.37	(0.65–2.89)	13	7	1.39	(0.66–2.95)
	Type 2	124	55	1.02	(0.77–1.34)	123	54	1.00	(0.75–1.33)

HR: hazard ratio; CI: confidence interval; BMI: body mass index.

[a] Defined as a new histologically confirmed tumour in the urinary bladder or prostatic urethra, after ≥1 tumour-negative follow-up cystoscopy result or a radical re-transurethral resection of the primary tumour.

[b] For the associations of BMI classes and obesity with recurrence, the adjustment set consists of age at time of UBC diagnosis, gender, highest completed level of education, weekly duration of physical activity, history of urinary bladder cancer among first degree relatives, tumour stage, tumour grade, and presence of concomitant CIS; for the associations of diabetes mellitus and type of diabetes mellitus with recurrence, the adjustment set consists of age at time of UBC diagnosis, gender, BMI classes, tumour stage, tumour grade, and presence of concomitant CIS.

[c] Number of incident events in the first 5 years after diagnosis of the primary non-muscle invasive urinary bladder cancer.

[d] Based on adult BMI; obesity was defined as a BMI ≥30.0 kg/m^2^, no obesity as a BMI <30.0 kg/m^2^.

[e] At the time of filling out the questionnaire; type 1 diabetes mellitus was defined as a diagnosis at age ≤30 years, or at age 31–40 years in combination without being obese, type 2 diabetes mellitus was defined as a diagnosis at age >40 years, or at age 31–40 years in combination with being obese.

### Associations of adult BMI and DM with progression

Tumour progression was less frequent compared to tumour recurrence: 170 patients experienced progression in the first 5 years after diagnosis, 44 of whom experienced progression to MIBC. Five-year risks of 14% (95% CI: 12%-16%) and 4% (95% CI: 3%-4%) were calculated for overall progression and progression to MIBC, respectively.

BMI, DM, DM1, and DM2 had no statistically significant association with risk of overall progression or progression to MIBC (S3 and S4 Figs and Table 3). For BMI and risk of overall progression, the proportional hazards assumption was not fulfilled, i.e. the risk of overall progression for obesity compared to healthy weight seemed to be decreased up to about 3.5 years of follow-up and to be increased afterwards (S3 Fig). The average five-year adjusted HRs for overweight and obesity, with healthy weight as reference, were 1.04 (95% CI: 0.74–1.44) and 1.20 (95% CI: 0.69–2.09), respectively. The adjusted HR for DM compared with no DM was 1.16 (95% CI: 0.76–1.76). Regarding progression to MIBC, the analyses for overweight and obesity yielded adjusted HRs of 0.84 (95% CI: 0.43–1.63) and 0.93 (95% CI: 0.27–3.16), respectively. The adjusted HR for DM was 1.40 (95% CI: 0.64–3.07). BMI as continuous variable also showed no associations with overall progression or progression to MIBC, with adjusted HRs per 1 kg/m^2^ increase of 0.99 (95% CI: 0.95–1.04) and 0.94 (95% CI: 0.84–1.05), respectively.

**Table 3:** Crude and adjusted hazard ratios (HR) with corresponding 95% confidence intervals (CI) for the associations of adult BMI and diabetes mellitus with overall progression ^a)^ and progression to Muscle Invasive Bladder Cancer (MIBC) ^b)^ among non-muscle invasive bladder cancer patients.

		*Crude analyses*				*Adjusted analyses* ^c)^			
		Number at risk	Events ^d)^	HR	(95% CI)	Number at risk	Events ^d)^	HR	(95% CI)
**Associations with overall progression**									
BMI classes in kg/m^2^ ^e)^									
	≥18.5 and <25.0	644	75	Reference		623	74	Reference	
	≥25.0 and <30.0	642	77	1.02	(0.74–1.40)	621	73	1.04	(0.74–1.44)
	≥30.0	128	16	1.08	(0.63–1.86)	125	16	1.20	(0.69–2.09)
Continuous BMI in kg/m^2^ ^e)^		1414	168	0.99	(0.95–1.04)	1369	163	0.99	(0.95–1.04)
Obesity ^e)^									
	No	1288	152	Reference		1245	147	Reference	
	Yes	128	16	1.07	(0.64–1.79)	125	16	1.18	(0.70–1.99)
Diabetes mellitus status ^f)^									
	No	1235	143	Reference		1201	139	Reference	
	Yes	198	27	1.16	(0.77–1.75)	191	27	1.16	(0.76–1.76)
Diabetes mellitus type ^f)^									
	No diabetes mellitus	1235	143	Reference		1201	139	Reference	
	Type 1	13	1	0.68	(0.10–4.84)	13	1	0.88	(0.12–6.36)
	Type 2	124	16	1.09	(0.65–1.83)	123	16	1.02	(0.60–1.72)
**Associations with progression to MIBC**									
BMI classes in kg/m^2^ ^e)^									
	≥18.5 and <25.0	644	22	Reference		623	21	Reference	
	≥25.0 and <30.0	642	18	0.81	(0.44–1.51)	621	16	0.84	(0.43–1.63)
	≥30.0	128	3	0.69	(0.21–2.31)	125	3	0.93	(0.27–3.16)
Continuous BMI in kg/m^2^ ^e)^		1414	43	0.94	(0.85–1.05)	1369	40	0.94	(0.84–1.05)
Obesity ^e)^									
	No	1286	40	Reference		1244	37	Reference	
	Yes	128	3	0.76	(0.24–2.47)	125	3	1.01	(0.31–3.31)
Diabetes mellitus status ^f)^									
	No	1235	36	Reference		1201	34	Reference	
	Yes	198	8	1.37	(0.64–2.94)	191	8	1.40	(0.64–3.07)
Diabetes mellitus type ^f)^									
	No diabetes mellitus	1235	36	Reference		1201	34	Reference	
	Type 1	13	1	2.82	(0.39–20.55)	13	1	5.65	(0.73–43.44)
	Type 2	124	5	1.35	(0.53–3.43)	123	5	1.24	(0.48–3.22)

HR: hazard ratio; CI: confidence interval; BMI: body mass index; MIBC: muscle invasive bladder cancer.

[a] Defined as the first occurrence of stage or grade progression, local or distant metastasis, and cystectomy for therapy-resistant disease.

[b] Defined as transition to MIBC (stage ≥T2).

[c] For the associations of BMI classes and obesity with progression, the adjustment set consists of age at time of UBC diagnosis, gender, highest completed level of education, weekly duration of physical activity, history of urinary bladder cancer among first degree relatives, tumour stage, tumour grade, and presence of concomitant CIS; for the associations of diabetes mellitus and type of diabetes mellitus with progression, the adjustment set consists of age at time of UBC diagnosis, gender, BMI classes, tumour stage, tumour grade, and presence of concomitant CIS.

[d] Number of incident events in the first 5 years after diagnosis of the primary non-muscle invasive urinary bladder cancer.

[e] Based on adult BMI; obesity was defined as a BMI ≥30.0 kg/m^2^, no obesity as a BMI <30.0 kg/m^2^.

[f] At the time of filling out the questionnaire; type 1 diabetes mellitus was defined as a diagnosis at age ≤30 years, or at age 31–40 years in combination without being obese, type 2 diabetes mellitus was defined as a diagnosis at age >40 years, or at age 31–40 years in combination with being obese.

## Discussion

In our large population-based cohort of NMIBC patients, we showed that neither adult BMI nor DM showed clear associations with risk of recurrence, overall progression or progression to MIBC. Previous studies on BMI and DM in relation to NMIBC prognosis are limited and results are inconsistent.

Four historical cohort studies investigated the association of overweight and/or obesity with the risk of recurrence and progression in NMIBC patients [19–21, 39]. In a study of 469 NMIBC patients, Xu et al. (2015) compared overweight and obesity to healthy weight at the time of transurethral resection of the bladder tumour. For obesity, HRs of 1.71 (95% CI: 1.12–2.60) and 3.04 (95% CI: 1.24–7.42) were found for recurrence and progression, respectively [21]. In a study of 892 patients with high grade T1 tumours by Kluth et al. (2013), timing of obesity was not reported. Here, statistically significant associations with recurrence (HR; 2.66, 95% CI: 2.13–3.32) and progression (HR: 1.49, 95% CI: 1.00–2.21) were shown when comparing obesity with no obesity [19]. In a recent study by Ferro et al. in 1155 T1G3 NMIBC patients treated with maintenance Bacillus Calmette-Guerin (BCG), obesity versus normal weight at diagnosis was associated with an increased risk of recurrence (HR: 5.33; 95% CI: 4.16–6.83) and progression (HR:2.51, 95% CI: 1.76–3.57) as well, with similar but slightly lower risk estimates for overweight [39]. None of these three studies adjusted for smoking status. A study by Wyszynski et al. (2014) including 726 NMIBC patients did adjust for smoking status. Here, no statistically significant association with risk of recurrence (HR: 1.22, 95% CI: 0.80–1.87) was reported when comparing obese to healthy weight patients at time of diagnosis [20]. We conducted the largest population-based cohort on this topic to date and, in contrast to the previous studies, investigated average BMI during adult life. This reflects lifetime exposure to growth factors and pro-inflammatory factors and is not affected by possible weight changes due to cancer diagnosis. In our study, no statistically significant association of adult BMI with risk of recurrence and progression was found. Smoking status and cigarette pack years were included in our DAG but not in our minimal sets of sufficient adjustment, because tests on the DAG showed that there was sufficient adjustment for the effect of smoking by including the other covariables in our models. This was confirmed by the fact that a sensitivity analysis with additional adjustment for smoking status did not change our risk estimates (data not shown). Also, no associations were found in the subgroup of patients initially treated with adjuvant immunotherapy (S5 Table), in contrast to results of Kluth et al. [19] and Ferro et al. [39]. Our results are in line with those of Wyszynski et al. [20], who also found that associations for BMI did not differ by immunotherapy treatment status. However, power for our sensitivity analysis was limited.

In a historical cohort of 251 NMIBC patients, Hwang et al. (2011) classified diagnosis of DM based on treatment history and fasting glucose levels. When comparing DM with no DM, they found HRs of 2.11 (95% CI: 1.4–3.2) and 9.35 (95% CI: 3.1–28.6) for recurrence and progression, respectively [22]. Rieken et al. (2013) retrieved information on DM diagnosis from medical records in a historical cohort study of 1117 NMIBC patients, and stratified their analyses for use of metformin [23]. When comparing DM without use of metformin with no DM, they found HRs of 1.39 (95% CI: 1.04–1.86) and 2.21 (95% CI: 1.29–3.77) for recurrence and progression, respectively. For DM with use of metformin compared with no DM, HRs of 0.48 (95% CI: 0.26–0.89) and 0.34 (95% CI: 0.05–2.42) were reported for recurrence and progression, respectively [23]. In a historical cohort of 645 NMIBC patients, Ahn et al. (2016) assessed DM diagnosis based on medical records, treatment history, and fasting glucose levels [24]. No statistically significant association of DM with recurrence (HR = 1.22; 95% CI: 0.89–1.67) or progression (HR = 1.54; 95% CI: 0.95–2.50) were found. Univariable analyses comparing diabetics who ever used metformin with diabetics who never used metformin yielded non-significant HRs of 1.07 (95% CI: 0.64–1.80) and 1.52 (95% CI: 0.70–3.33) for recurrence and progression, respectively [24]. In our study, patients were asked whether they were ever diagnosed with DM by a physician and at which age. No association with risk of recurrence or progression was found. Since treatment for DM was not assessed in our study, effect modification by DM treatment could not be evaluated.

Our study had several limitations. First, patients were invited and completed questionnaires relatively long after diagnosis, and information about BMI and DM was self-reported. Also, the possibility of recall errors cannot be excluded although we do not expect these errors to be different between patients with or without an event. Another limitation was that we could not perform analyses stratified for the timing of DM diagnosis relative to the events of interest, because 31% of the DM diagnosed patients had missing values for age at DM diagnosis. Thus, also patients with a known age at DM diagnosis and who indicated to be diagnosed with DM between diagnosis and time of filling out the questionnaire were included as diabetics. Since 27% of patients with available age at DM diagnosis were diagnosed after NMIBC diagnosis, this may have influenced our results. Patients who had missing values for DM diagnosis were included as having no DM. To validate, we performed sensitivity analyses by comparing the study results with the results of two alternative methods to deal with missing DM status. In the first alternative, we assumed that patients only filled out questions on medical conditions with which they were diagnosed. Patients with missing DM status were only included as non-diabetics if they never answered negatively to a question regarding medical history and they answered positively to at least one medical history-question. In the second alternative, patients with missing values for DM diagnosis were excluded by default. Reassuringly, all methods resulted in similar findings (S3 and S4 Tables). Another limitation was the lack of information on DM type. We therefore used age at diagnosis and BMI to classify into DM1 and DM2. This may have caused some misclassification. Besides, diabetics who did not report age at diagnosis could not be classified and had to be excluded from the stratified analyses. Further, we could not draw conclusions from our study regarding changes in BMI up to or after diagnosis. Also, the possibility of residual confounding could not be excluded. Finally, we had limited power for the analyses of progression to MIBC due to a limited number of events, and as shown by the wide confidence intervals for these risk estimates.

Strengths of our study compared with previous studies are the inclusion of a large population-based series of patients. Since NMIBC patients included in the current analysis did not differ from invited NMIBC patients with respect to age, gender, tumour stage, and tumour grade (data not shown), our results are unlikely to have been affected by selection bias. Moreover, the five-year risks of recurrence and progression in our population are in line with estimates in the literature [4]. Also, we used a state-of-the-art approach in selecting the adjustment sets. By use of the DAG, minimal sets for sufficient adjustment were selected. Because over-adjustment was prevented, we achieved better precision in estimating the effect.

In conclusion, we did not find associations of adult BMI and DM with risk of recurrence and progression among NMIBC patients in the first five years after diagnosis. Considering the designs and results of our and previous studies, and their limitations, we cannot draw definitive conclusions. To obtain more insight in the associations of overweight and obesity, including weight change, and DM with risk of recurrence and progression in NMIBC patients, we recommend the conduction of prospective cohort studies assessing BMI, DM type and medication both before and after NMIBC diagnosis. Results of such studies may contribute to the development of personalized follow-up schedules and may also aid in advising NMIBC patients about weight and DM management to improve their prognosis.

## Supporting information

S1 Fig Directed Acyclic Graph for the associations of BMI and diabetes mellitus with urinary bladder cancer recurrence and progression. (TIF)

S2 Fig Cumulative risk plots for recurrence, stratified by BMI class [A] and diabetes mellitus status [B].
(TIF)

S3 Fig Cumulative risk plots for overall progression [A] and progression to muscle invasive bladder cancer [B], stratified by BMI class.
(TIF)

S4 Fig Cumulative risk plots for overall progression [A] and progression to muscle invasive bladder cancer [B], stratified by diabetes mellitus (DM) status.
(TIF)

S1 Table Patient characteristics on lifestyle and medical history, and characteristics regarding their primary bladder tumour and initial treatment, stratified by BMI classes. P25: 25th percentile; P75: 75th percentile; BMI: body mass index; CIS: carcinoma in situ; TURT: transurethral resection of the bladder tumour. [a] At the time of non-muscle invasive bladder cancer diagnosis. [b] Based on the country of birth of the patients and their parents. [c] Based on the International Standard Classification of Education: low level includes elementary, lower vocational and intermediate general education; intermediate level includes intermediate vocational and higher general education; high level includes higher vocational education and university. [d] Presented for current and former cigarette smokers. [e] Based on the weekly duration of walking, cycling, and sporting during adult life until 2 years before diagnosis; unknown for 25 patients (1.7%). [f] At the time of filling out the questionnaire. [g] 66 patients (4.6%) had a missing value for diagnosis of diabetes mellitus and were included as not diagnosed. [h] Presented for patients diagnosed with diabetes mellitus; type 1 was defined as diabetes mellitus diagnosis at age ≤30 years, or at age 31–40 years in combination with being not obese; type 2 was defined as diabetes mellitus diagnosis at age >40 years, or at age 31–40 years in combination with being obese. [i] Tumours with WHO 1973 differentiation grade 1 or 2, WHO/ISUP 2004 low grade, or Malmström (Modified Bergkvist) grade 1 or 2a were considered low-grade tumours. Tumours with WHO 1973 differentiation grade 3, WHO/ISUP 2004 high grade, or Malmström (Modified Bergkvist) grade 2b or 3 as high-grade. [j] Based on the European Association of Urology guidelines: low risk includes Ta-stage with low grade, high risk includes Ta-stage with high grade, T1-stage, T2-stage, T3-stage, T4-stage, and CIS.
(DOCX)

S2 Table Patient characteristics on lifestyle and medical history, and characteristics regarding their primary bladder tumour and initial treatment, stratified by diabetes mellitus exposure groups. P25: 25th percentile; P75: 75th percentile; BMI: body mass index; CIS: carcinoma in situ; DM: diabetes mellitus; TURT: transurethral resection of the bladder tumour. [a] 66 patients (4.6%) had a missing value for diagnosis of diabetes mellitus and were included as not diagnosed. [b] Type 1 was defined as diabetes mellitus diagnosis at age ≤30 years, or at age 31–40 years in combination with being not obese; type 2 was defined as diabetes mellitus diagnosis at age >40 years, or at age 31–40 years in combination with being obese. [c] At the time of non-muscle invasive bladder cancer diagnosis. [d] Based on the country of birth of the patients and their parents. [e] Based on the International Standard Classification of Education: low level includes elementary, lower vocational and intermediate general education; intermediate level includes intermediate vocational and higher general education; high level includes higher vocational education and university. [f] Presented for current and former cigarette smokers. [g] Based on the weekly duration of walking, cycling, and sporting during adult life until 2 years before diagnosis; unknown for 25 patients (1.7%). [h] At the time of filling out the questionnaire. [i] Tumours with WHO 1973 differentiation grade 1 or 2, WHO/ISUP 2004 low grade, or Malmström (Modified Bergkvist) grade 1 or 2a were considered low-grade tumours. Tumours with WHO 1973 differentiation grade 3, WHO/ISUP 2004 high grade, or Malmström (Modified Bergkvist) grade 2b or 3 as high-grade. [j] Based on the European Association of Urology guidelines: low risk includes Ta-stage with low grade, high risk includes Ta-stage with high grade, T1-stage, T2-stage, T3-stage, T4-stage, and CIS.
(DOCX)

S3 Table Crude and adjusted hazard ratios (HR) with corresponding 95% confidence intervals (CI) for the association of diabetes mellitus with recurrence ^a)^ among non-muscle invasive bladder cancer patients, using different methods for dealing with missing values on diabetes mellitus diagnosis. HR: hazard ratio; CI: confidence interval. [a] Defined as a new histologically confirmed tumour in the urinary bladder or prostatic urethra, after ≥1 tumour-negative follow-up cystoscopy result or a radical re-transurethral resection of the primary tumour. [b] The adjustment set consists of age at time of UBC diagnosis, gender, BMI classes, tumour stage, tumour grade, and presence of concomitant CIS. [c] Number of events in the first 5 years after diagnosis of the primary non-muscle invasive urinary bladder cancer. [d] At the time of filling out the questionnaire.
(DOCX)

S4 Table Crude and adjusted hazard ratios (HR) with corresponding 95% confidence intervals (CI) for the association of diabetes mellitus with progression among non-muscle invasive bladder cancer patients, using different methods for dealing with missing values on diabetes mellitus diagnosis. HR: hazard ratio; CI: confidence interval; MIBC: muscle invasive bladder cancer. [a] The adjustment set consists of age at time of UBC diagnosis, gender, BMI classes, tumour stage, S5 Table. Crude and adjusted hazard ratios (HR) with corresponding 95% confidence intervals (CI) for the associations of BMI with recurrence, overall progression, and progression to MIBC, among non-muscle invasive bladder cancer patients who received transurethral resection of the bladder tumour (TURT) with one intravesical chemotherapy instillation and adjuvant intravesical immunotherapy. [b] Number of events in the first 5 years after diagnosis of the primary non-muscle invasive urinary bladder cancer. [c] Defined as the first occurrence of stage or grade progression, local or distant metastasis, and cystectomy for therapy-resistant disease. [d] At the time of filling out the questionnaire. [e] Defined as transition to MIBC (stage ≥T2).
(DOCX)

S5 Table Crude and adjusted hazard ratios (HR) with corresponding 95% confidence intervals (CI) for the associations of BMI with recurrence, overall progression, and progression to MIBC, among non-muscle invasive bladder cancer patients who received transurethral resection of the bladder tumour (TURT) with one intravesical chemotherapy instillation and adjuvant intravesical immunotherapy. HR: hazard ratio; CI: confidence interval; BMI: body mass index. [a] Defined as a new histologically confirmed tumour in the urinary bladder or prostatic urethra, after ≥1 tumour-negative follow-up cystoscopy result or a radical re-transurethral resection of the primary tumour. [b] Defined as the first occurrence of stage or grade progression, local or distant metastasis, and cystectomy for therapy-resistant disease [c] Defined as transition to MIBC (stage ≥T2). [d] The adjustment set consists of age at time of UBC diagnosis, gender, highest completed level of education, weekly duration of physical activity, history of urinary bladder cancer among first degree relatives, tumour stage, tumour grade, and presence of concomitant CIS. [e] Number of incident events in the first 5 years after diagnosis of the primary non-muscle invasive urinary bladder cancer. [f] Based on average BMI during adult life.
(DOCX)
