# Supplementary figures and images for: No clear associations of adult BMI and diabetes mellitus with non-muscle invasive bladder cancer recurrence and progression
Source: PLoS One. 2020 Mar 25;15(3):e0229384. doi: 10.1371/journal.pone.0229384 (PMC7094867; doi:10.1371/journal.pone.0229384)

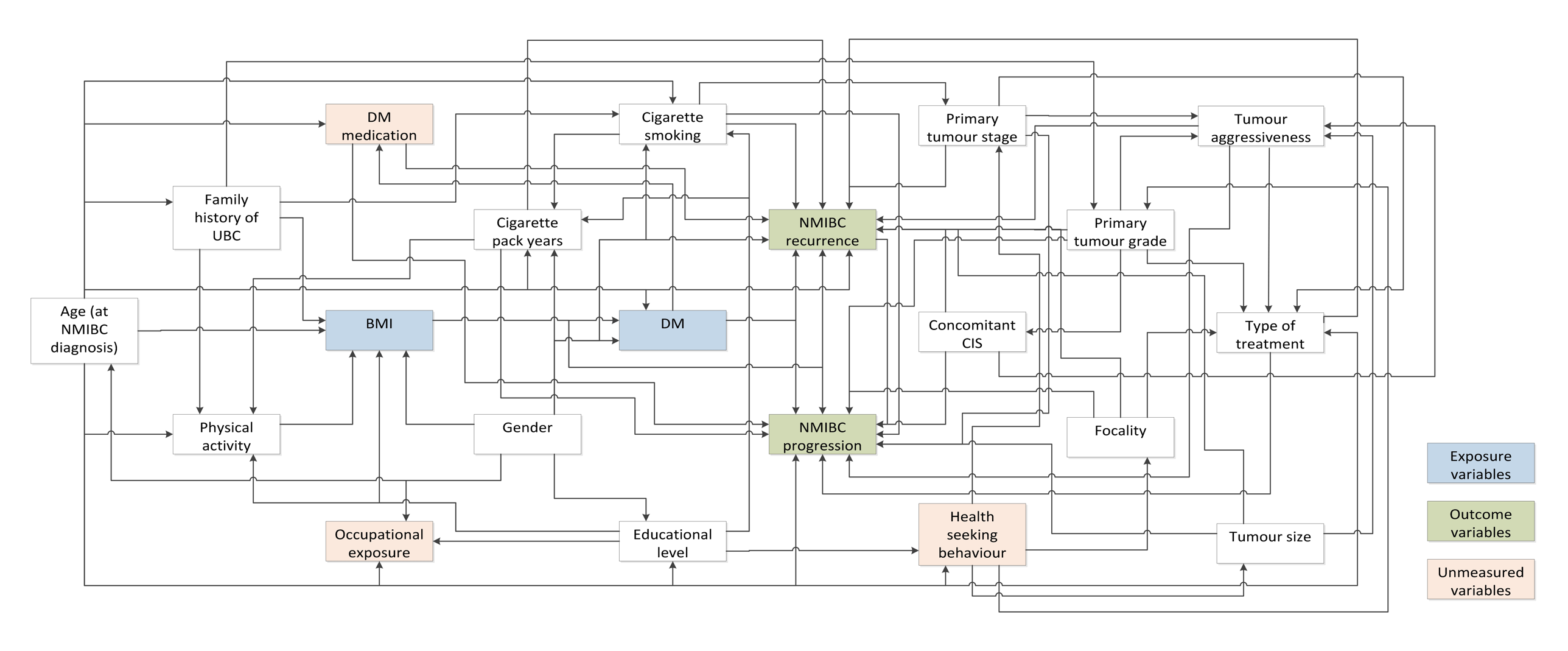

Supplement: S1 Fig — (TIF) [file pone.0229384.s001.tif]

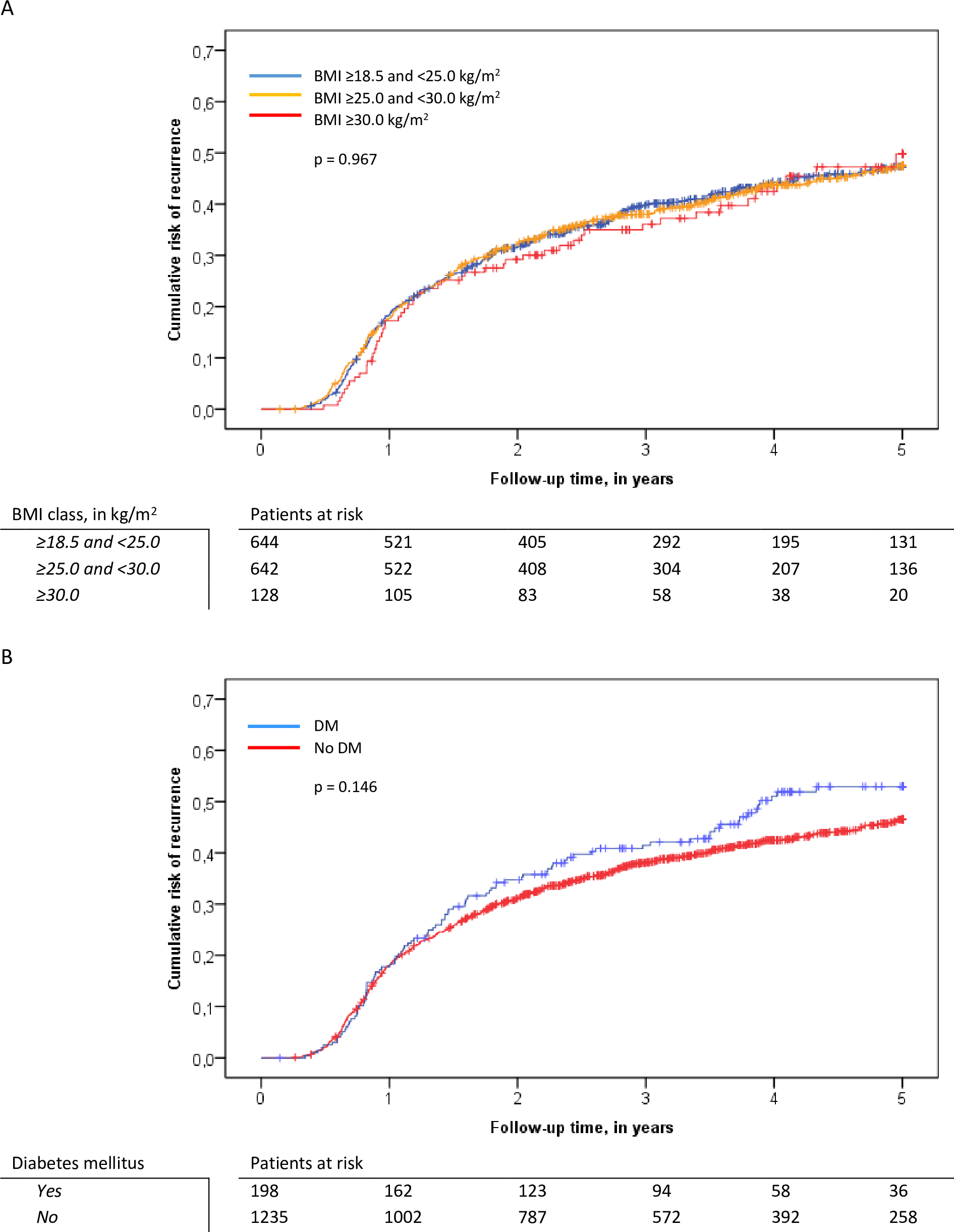

Supplement: S2 Fig — Cumulative risk plots for recurrence, stratified by BMI class [A] and diabetes mellitus status [B]. (TIF) [file pone.0229384.s002.tif]

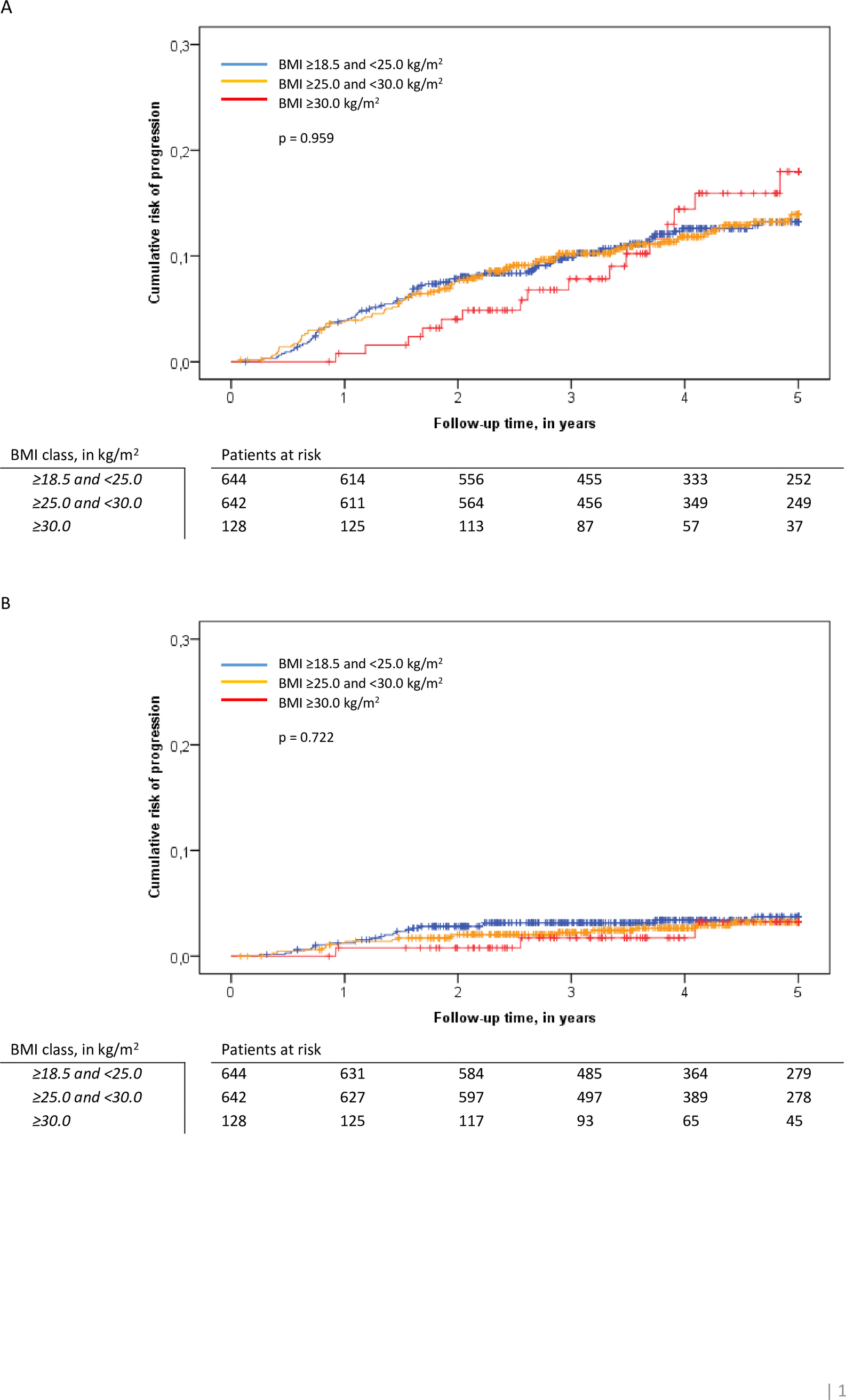

Supplement: S3 Fig — Cumulative risk plots for overall progression [A] and progression to muscle invasive bladder cancer [B], stratified by BMI class. (TIF) [file pone.0229384.s003.tif]

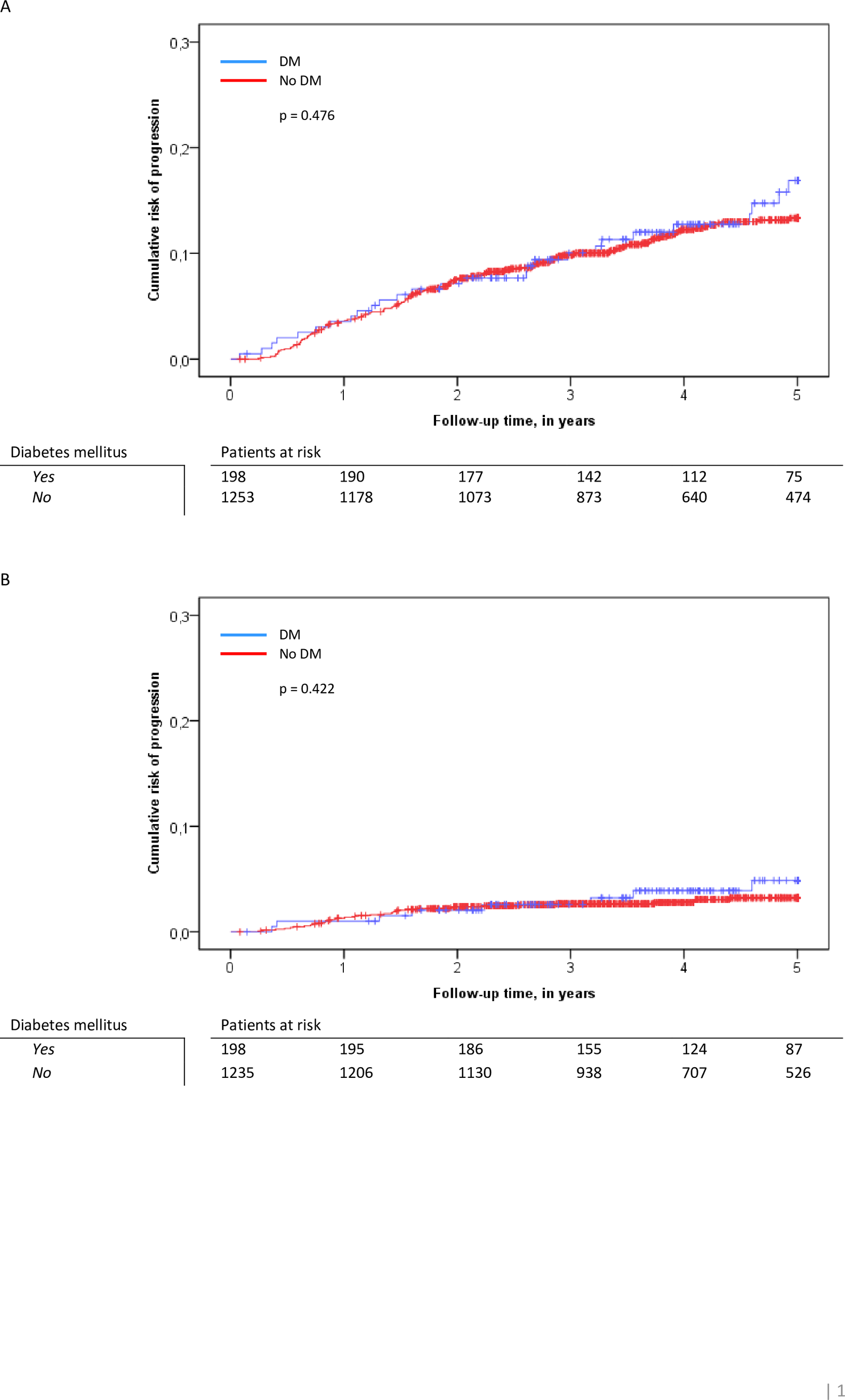

Supplement: S4 Fig — Cumulative risk plots for overall progression [A] and progression to muscle invasive bladder cancer [B], stratified by diabetes mellitus (DM) status. (TIF) [file pone.0229384.s004.tif]
