# Supplementary material for: No clear associations of adult BMI and diabetes mellitus with non-muscle invasive bladder cancer recurrence and progression
Source: PLoS One. 2020 Mar 25;15(3):e0229384. doi: 10.1371/journal.pone.0229384 (PMC7094867; doi:10.1371/journal.pone.0229384)
Supplement: S1 Table — P25: 25th percentile; P75: 75th percentile; BMI: body mass index; CIS: carcinoma in situ; TURT: transurethral resection of the bladder tumour. [a] At the time of non-muscle invasive bladder cancer diagnosis. [b] Based on the country of birth of the patients and their parents. [c] Based on the International Standard Classification of Education: low level includes elementary, lower vocational and intermediate general education; intermediate level includes intermediate vocational and higher general education; high level includes higher vocational education and university. [d] Presented for current and former cigarette smokers. [e] Based on the weekly duration of walking, cycling, and sporting during adult life until 2 years before diagnosis; unknown for 25 patients (1.7%). [f] At the time of filling out the questionnaire. [g] 66 patients (4.6%) had a missing value for diagnosis of diabetes mellitus and were included as not diagnosed. [h] Presented for patients diagnosed with diabetes mellitus; type 1 was defined as diabetes mellitus diagnosis at age ≤30 years, or at age 31–40 years in combination with being not obese; type 2 was defined as diabetes mellitus diagnosis at age >40 years, or at age 31–40 years in combination with being obese. [i] Tumours with WHO 1973 differentiation grade 1 or 2, WHO/ISUP 2004 low grade, or Malmström (Modified Bergkvist) grade 1 or 2a were considered low-grade tumours. Tumours with WHO 1973 differentiation grade 3, WHO/ISUP 2004 high grade, or Malmström (Modified Bergkvist) grade 2b or 3 as high-grade. [j] Based on the European Association of Urology guidelines: low risk includes Ta-stage with low grade, high risk includes Ta-stage with high grade, T1-stage, T2-stage, T3-stage, T4-stage, and CIS. (DOCX) [file pone.0229384.s005.docx]

| **S1 Table. Patient characteristics on lifestyle and medical history, and characteristics regarding their primary bladder tumour and initial treatment, stratified by BMI classes** | | | | | | | | | | |
| --- | --- | --- | --- | --- | --- | --- | --- | --- | --- | --- |
|  | | | **BMI ≥18.5 and <25.0 kg/m^2^ (N = 644)** | | **BMI ≥25.0 and <30.0 kg/m^2^**  **(N = 642)** | | **BMI ≥30.0 kg/m^2^ [obese]**  **(N = 128)** | | **BMI <30.0 kg/m^2^ [not obese]**  **(N = 1286)** | |
|  | | | n | (%) | n | (%) | n | (%) | n | (%) |
| **Demographic characteristics** | | | | | | | | | | |
| Age in years, median (P_25_, P_75_) ^a)^ | | | 63.0 | (56.0, 70.0) | 64.0 | (57.0, 70.0) | 62.0 | (56.0, 68.0) | 64.0 | (56.0, 70.0) |
| Male | | | 507 | (78.7) | 566 | (88.2) | 100 | (78.1) | 1073 | (83.4) |
| Ethnicity ^b)^ | | | | | | | | | | |
|  | Dutch | | 574 | (89.1) | 587 | (91.4) | 114 | (89.1) | 1161 | (90.3) |
|  | Non-Dutch | | 70 | (10.9) | 55 | (8.6) | 14 | (10.9) | 125 | (9.7) |
| Highest completed level of education ^c)^ | | | | | | | | | | |
|  | Low | | 314 | (48.8) | 374 | (58.3) | 93 | (72.7) | 688 | (53.5) |
|  | Intermediate | | 146 | (22.7) | 134 | (20.9) | 24 | (18.8) | 280 | (21.8) |
|  | High | | 182 | (28.3) | 134 | (20.9) | 11 | (8.6) | 316 | (24.5) |
|  | Unknown | | 2 | (0.3) | 0 | (0.0) | 0 | (0.0) | 2 | (0.2) |
| **Lifestyle factors** | | | | | | | | | | |
| Cigarette smoking status ^a)^ | | | | | | | | | | |
|  | Current | | 243 | (37.7) | 236 | (36.8) | 45 | (35.2) | 479 | (37.2) |
|  | Former | | 267 | (41.5) | 290 | (45.2) | 58 | (45.3) | 557 | (43.3) |
|  | Never | | 126 | (19.6) | 106 | (16.5) | 22 | (17.2) | 232 | (18.0) |
|  | Unknown | | 8 | (1.2) | 10 | (1.6) | 3 | (2.3) | 18 | (1.4) |
| Cigarette pack-years, median (P_25_, P_75_) ^a) d)^ | | | 22.0 | (10.0, 35.0) | 22.0 | (12.0, 35.0) | 25.0 | (15.0, 38.8) | 22.0 | (12.0, 35.0) |
| Weekly duration of physical activity in hours, median (P_25_, P_75_) ^e)^ | | | 10.0 | (6.0, 15.5) | 8.5 | (5.0, 14.0) | 7.0 | (4.0, 14.0) | 9.0 | (5.5, 15.0) |
| **Medical history** | | | | | | | | | | |
| Diagnosed with diabetes mellitus ^f)^ | | | | | | | | | | |
|  | Yes | | 59 | (9.2) | 95 | (14.8) | 41 | (32.0) | 154 | (88.0) |
|  | No ^g)^ | | 585 | (90.8) | 547 | (85.2) | 87 | (68.0) | 1132 | (12.0) |
| Diabetes mellitus type ^f) h)^ | | | | | | | | | | |
|  | Type 1 | | 3 | (5.1) | 10 | (10.5) | 0 | (0.0) | 13 | (8.4) |
|  | Type 2 | | 39 | (66.1) | 54 | (56.8) | 31 | (75.6) | 93 | (60.4) |
|  | Unknown | | 17 | (28.8) | 31 | (32.6) | 10 | (24.4) | 48 | (31.2) |
| UBC history among first degree relatives ^f)^ | | | | | | | | | | |
|  | Yes | | 44 | (6.8) | 46 | (7.2) | 7 | (5.5) | 90 | (7.0) |
|  | No | | 600 | (93.2) | 596 | (92.8) | 121 | (94.5) | 1196 | (93.0) |
| **Primary tumour characteristics** | | | | | | | | | | |
| Stage, TNM 2002 classification | | | | | | | | | | |
|  | Ta | | 450 | (69.9) | 451 | (70.2) | 96 | (75.0) | 901 | (70.1) |
|  | CIS | | 19 | (3.0) | 29 | (4.5) | 4 | (3.1) | 48 | (3.7) |
|  | T1 | | 166 | (25.8) | 150 | (23.4) | 27 | (21.1) | 316 | (24.6) |
|  | Unknown | | 9 | (1.4) | 12 | (1.9) | 1 | (0.8) | 21 | (1.6) |
| Grade ^i)^ | | | | | | | | | | |
|  | Low grade (G1 or G2) | | 420 | (65.2) | 406 | (63.2) | 85 | (66.4) | 826 | (64.2) |
|  | High grade (G3) | | 219 | (34.0) | 227 | (35.4) | 41 | (32.0) | 446 | (34.7) |
|  | Unknown | | 5 | (0.8) | 9 | (1.4) | 2 | (1.6) | 14 | (1.1) |
| Risk of progression ^j)^ | | | | | | | | | | |
|  | Low | | 382 | (59.3) | 371 | (57.8) | 77 | (60.2) | 753 | (58.6) |
|  | High | | 255 | (39.6) | 260 | (40.5) | 49 | (38.3) | 515 | (40.0) |
|  | Unknown | | 7 | (1.1) | 11 | (1.7) | 2 | (1.6) | 18 | (1.4) |
| Histology | | | | | | | | | | |
|  | Urothelial cell carcinoma | | 641 | (99.5) | 637 | (99.2) | 127 | (99.2) | 1278 | (99.4) |
|  | Other, including combinations of histology types | | 2 | (0.3) | 0 | (0.0) | 0 | (0.0) | 2 | (0.2) |
|  | Unknown | | 1 | (0.2) | 5 | (0.8) | 1 | (0.8) | 6 | (0.5) |
| Initial treatment | | | | | | | | | | |
|  | TURT with one intravesical chemotherapy instillation | | 304 | (47.2) | 293 | (45.6) | 67 | (52.3) | 597 | (46.4) |
|  | Adjuvant intravesical chemotherapy | | 187 | (29.0) | 212 | (33.0) | 33 | (25.8) | 399 | (31.0) |
|  | Adjuvant intravesical immunotherapy | | 123 | (19.1) | 111 | (17.3) | 23 | (18.0) | 234 | (18.2) |
|  | Adjuvant intravesical chemo- and immunotherapy | | 7 | (1.1) | 8 | (1.2) | 3 | (2.3) | 15 | (1.2) |
|  | Other | | 0 | (0.0) | 1 | (0.2) | 0 | (0.0) | 1 | (0.1) |
|  | Unknown | | 23 | (3.6) | 17 | (2.6) | 2 | (1.6) | 40 | (3.1) |
| Concomitant CIS | | | | | | | | | | |
|  | Yes | | 54 | (8.4) | 40 | (6.2) | 10 | (7.8) | 94 | (7.3) |
|  | No | | 584 | (90.7) | 591 | (92.1) | 117 | (91.4) | 1175 | (91.4) |
|  | Unknown | | 6 | (0.9) | 11 | (1.7) | 1 | (0.8) | 17 | (1.3) |
| Focality | | | | | | | | | | |
|  | Unifocal | | 363 | (56.4) | 343 | (53.4) | 71 | (55.5) | 706 | (54.9) |
|  | Multifocal | | 243 | (37.7) | 264 | (41.1) | 49 | (38.3) | 507 | (39.4) |
|  | Unknown | | 38 | (5.9) | 35 | (5.5) | 8 | (6.3) | 73 | (5.7) |
| Size in cm | | | | | | | | | | |
|  | <3 | | 91 | (14.1) | 83 | (12.9) | 18 | (14.1) | 174 | (13.5) |
|  | ≥3 | | 41 | (6.4) | 54 | (8.4) | 15 | (11.7) | 95 | (7.4) |
|  | Unknown | | 512 | (79.5) | 505 | (78.7) | 95 | (74.2) | 1017 | (79.1) |
| P_25_: 25^th^ percentile; P_75_: 75^th^ percentile; BMI: body mass index; CIS: carcinoma in situ; TURT: transurethral resection of the bladder tumour | | | | | | | | | | |
| [a] | | At the time of non-muscle invasive bladder cancer diagnosis | | | | | | | | |
| [b] | | Based on the country of birth of the patients and their parents | | | | | | | | |
| [c] | | Based on the International Standard Classification of Education: low level includes elementary, lower vocational and intermediate general education; intermediate level includes intermediate vocational and higher general education; high level includes higher vocational education and university | | | | | | | | |
| [d] | | Presented for current and former cigarette smokers | | | | | | | | |
| [e] | | Based on the weekly duration of walking, cycling, and sporting during adult life until 2 years before diagnosis; unknown for 25 patients (1.7%) | | | | | | | | |
| [f] | | At the time of filling out the questionnaire | | | | | | | | |
| [g] | | 66 patients (4.6%) had a missing value for diagnosis of diabetes mellitus and were included as not diagnosed | | | | | | | | |
| [h] | | Presented for patients diagnosed with diabetes mellitus; type 1 was defined as diabetes mellitus diagnosis at age ≤30 years, or at age 31-40 years in combination with being not obese; type 2 was defined as diabetes mellitus diagnosis at age >40 years, or at age 31-40 years in combination with being obese | | | | | | | | |
| [i] | | Tumours with WHO 1973 differentiation grade 1 or 2, WHO/ISUP 2004 low grade, or Malmström (Modified Bergkvist) grade 1 or 2a were considered low-grade tumours. Tumours with WHO 1973 differentiation grade 3, WHO/ISUP 2004 high grade, or Malmström (Modified Bergkvist) grade 2b or 3 as high-grade | | | | | | | | |
| [j] | | Based on the European Association of Urology guidelines: low risk includes Ta-stage with low grade, high risk includes Ta-stage with high grade, T1-stage, T2-stage, T3-stage, T4-stage, and CIS | | | | | | | | |
