# Supplementary material for: No clear associations of adult BMI and diabetes mellitus with non-muscle invasive bladder cancer recurrence and progression
Source: PLoS One. 2020 Mar 25;15(3):e0229384. doi: 10.1371/journal.pone.0229384 (PMC7094867; doi:10.1371/journal.pone.0229384)
Supplement: S2 Table — P25: 25th percentile; P75: 75th percentile; BMI: body mass index; CIS: carcinoma in situ; DM: diabetes mellitus; TURT: transurethral resection of the bladder tumour. [a] 66 patients (4.6%) had a missing value for diagnosis of diabetes mellitus and were included as not diagnosed. [b] Type 1 was defined as diabetes mellitus diagnosis at age ≤30 years, or at age 31–40 years in combination with being not obese; type 2 was defined as diabetes mellitus diagnosis at age >40 years, or at age 31–40 years in combination with being obese. [c] At the time of non-muscle invasive bladder cancer diagnosis. [d] Based on the country of birth of the patients and their parents. [e] Based on the International Standard Classification of Education: low level includes elementary, lower vocational and intermediate general education; intermediate level includes intermediate vocational and higher general education; high level includes higher vocational education and university. [f] Presented for current and former cigarette smokers. [g] Based on the weekly duration of walking, cycling, and sporting during adult life until 2 years before diagnosis; unknown for 25 patients (1.7%). [h] At the time of filling out the questionnaire. [i] Tumours with WHO 1973 differentiation grade 1 or 2, WHO/ISUP 2004 low grade, or Malmström (Modified Bergkvist) grade 1 or 2a were considered low-grade tumours. Tumours with WHO 1973 differentiation grade 3, WHO/ISUP 2004 high grade, or Malmström (Modified Bergkvist) grade 2b or 3 as high-grade. [j] Based on the European Association of Urology guidelines: low risk includes Ta-stage with low grade, high risk includes Ta-stage with high grade, T1-stage, T2-stage, T3-stage, T4-stage, and CIS. (DOCX) [file pone.0229384.s006.docx]

| **S2 Table. Patient characteristics on lifestyle and medical history, and characteristics regarding their primary bladder tumour and initial treatment, stratified by diabetes mellitus exposure groups** | | | | | | | | | | |
| --- | --- | --- | --- | --- | --- | --- | --- | --- | --- | --- |
|  | | | **No DM ^a)^**  **(N = 1235)** | | **DM**  **(N = 198)** | | **DM1 ^b)^**  **(N = 13)** | | **DM2 ^b)^**  **(N = 124)** | |
|  | | | n | (%) | n | (%) | n | (%) | n | (%) |
| **Demographic characteristics** | | | | | | | | | | |
| Age in years, median (P_25_, P_75_) ^c)^ | | | 63.0 | (56.0, 70.0) | 65.0 | (60.0, 70.3) | 55.0 | (45.5, 62.0) | 65.0 | (60.0, 70.0) |
| Male | | | 1020 | (82.6) | 167 | (84.3) | 11 | (84.6) | 100 | (80.6) |
| Ethnicity ^d)^ | | | | | | | | | | |
|  | Dutch | | 1112 | (90.0) | 182 | (91.9) | 12 | (92.3) | 112 | (90.3) |
|  | Non-Dutch | | 123 | (10.0) | 16 | (8.1) | 1 | (7.7) | 12 | (9.7) |
| Highest completed level of education ^e)^ | | | | | | | | | | |
|  | Low | | 666 | (53.9) | 128 | (64.6) | 5 | (38.5) | 83 | (66.9) |
|  | Intermediate | | 268 | (21.7) | 40 | (20.2) | 4 | (30.8) | 28 | (22.6) |
|  | High | | 300 | (24.3) | 29 | (14.6) | 4 | (30.8) | 13 | (10.5) |
|  | Unknown | | 1 | (0.1) | 1 | (0.5) | 0 | (0.0) | 0 | (0.0) |
| **Lifestyle factors** | | | | | | | | | | |
| Average BMI during adult life in kg/m^2^ | | | | | | | | | | |
|  | <18.5 | | 2 | (0.2) | 0 | (0.0) | 0 | (0.0) | 0 | (0.0) |
|  | ≥18.5 and <25.0 | | 585 | (47.4) | 59 | (29.8) | 3 | (23.1) | 39 | (31.5) |
|  | ≥25.0 and <30.0 | | 547 | (44.3) | 95 | (48.0) | 10 | (76.9) | 54 | (43.5) |
|  | ≥30.0 | | 87 | (7.0) | 41 | (20.7) | 0 | (0.0) | 31 | (25.0) |
|  | Unknown | | 14 | (1.1) | 3 | (1.5) | 0 | (0.0) | 0 | (0.0) |
|  | Median (P_25_, P_75_) | | 25.1 | (23.7, 26.9) | 27.0 | (24.6, 29.4) | 26.9 | (23.1, 27.7) | 27 | (24.4, 30.0) |
| Cigarette smoking status ^c)^ | | | | | | | | | | |
|  | Current | | 466 | (37.7) | 65 | (32.8) | 5 | (38.5) | 41 | (33.1) |
|  | Former | | 528 | (42.8) | 92 | (46.5) | 6 | (46.2) | 54 | (43.5) |
|  | Never | | 227 | (18.4) | 32 | (16.9) | 2 | (15.4) | 22 | (17.7) |
|  | Unknown | | 14 | (1.1) | 9 | (4.5) | 0 | (0.0) | 7 | (5.6) |
| Cigarette pack-years, median (P_25_, P_75_) ^c) f)^ | | | 21.0 | (11.0, 34.0) | 27.0 | (18.0, 42.0) | 18.0 | (7.0, 30.0) | 27.0 | (19.0, 42.0) |
| Weekly duration of physical activity in hours, median (P_25_, P_75_) ^g)^ | | | 9.0 | (5.5, 15.0) | 8.3 | (4.0, 14.1) | 6.0 | (3.5, 13.3) | 8.5 | (4.0, 14.5) |
| **Medical history** | | | | | | | | | | |
| UBC history among first degree relatives ^h)^ | | | | | | | | | | |
|  | Yes | | 82 | (6.6) | 16 | (8.1) | 2 | (15.4) | 9 | (7.3) |
|  | No | | 1153 | (93.4) | 182 | (91.9) | 11 | (84.6) | 115 | (92.7) |
| **Primary tumour characteristics** | | | | | | | | | | |
| Stage, TNM 2002 classification | | | | | | | | | | |
|  | Ta | | 872 | (70.6) | 136 | (68.7) | 10 | (76.9) | 86 | (69.4) |
|  | CIS | | 48 | (3.9) | 6 | (3.0) | 0 | (0.0) | 4 | (3.2) |
|  | T1 | | 295 | (23.9) | 54 | (27.3) | 3 | (23.1) | 33 | (26.6) |
|  | Unknown | | 20 | (1.6) | 2 | (1.0) | 0 | (0.0) | 1 | (0.8) |
| Grade ^i)^ | | | | | | | | | | |
|  | Low grade (G1 or G2) | | 801 | (64.9) | 120 | (60.6) | 10 | (76.9) | 76 | (61.3) |
|  | High grade (G3) | | 422 | (34.2) | 74 | (37.4) | 3 | (23.1) | 47 | (37.9) |
|  | Unknown | | 12 | (1.0) | 3 | (2.0) | 0 | (0.0) | 1 | (0.8) |
| Risk of progression ^j)^ | | | | | | | | | | |
|  | Low | | 732 | (59.3) | 108 | (54.5) | 9 | (69.2) | 69 | (55.6) |
|  | High | | 486 | (39.4) | 87 | (43.9) | 4 | (30.8) | 54 | (43.5) |
|  | Unknown | | 17 | (1.4) | 3 | (1.5) | 0 | (0.0) | 1 | (0.8) |
| Histology | | | | | | | | | | |
|  | Urothelial cell carcinoma | | 1226 | (99.3) | 198 | (100.0) | 13 | (100.0) | 124 | (100.0) |
|  | Other, including combinations of histology types | | 2 | (0.2) | 0 | (0.0) | 0 | (0.0) | 0 | (0.0) |
|  | Unknown | | 7 | (0.6) | 0 | (0.0) | 0 | (0.0) | 0 | (0.0) |
| Initial treatment | | | | | | | | | | |
|  | TURT with one intravesical chemotherapy instillation | | 587 | (47.5) | 81 | (40.9) | 6 | (46.2) | 51 | (41.1) |
|  | Adjuvant intravesical lchemotherapy | | 378 | (30.6) | 65 | (32.8) | 4 | (30.8) | 42 | (33.9) |
|  | Adjuvant intravesical immunotherapy | | 219 | (17.7) | 42 | (21.2) | 2 | (15.4) | 27 | (21.8) |
|  | Adjuvant intravesical chemo- and immunotherapy | | 15 | (1.2) | 3 | (1.5) | 0 | (0.0) | 2 | (1.6) |
|  | Other | | 0 | (0.0) | 1 | (0.5) | 0 | (0.0) | 1 | (0.8) |
|  | Unknown | | 36 | (2.9) | 6 | (3.0) | 1 | (7.7) | 1 | (0.8) |
| Concomitant CIS | | | | | | | | | | |
|  | Yes | | 87 | (7.0) | 18 | (9.1) | 0 | (0.0) | 12 | (9.7) |
|  | No | | 1132 | (91.7) | 178 | (89.9) | 13 | (100.0) | 111 | (89.5) |
|  | Unknown | | 16 | (1.3) | 2 | (1.0) | 0 | (0.0) | 1 | (0.8) |
| Focality | | | | | | | | | | |
|  | Unifocal | | 674 | (54.6) | 112 | (56.6) | 10 | (76.9) | 69 | (55.6) |
|  | Multifocal | | 490 | (39.7) | 74 | (37.4) | 2 | (15.4) | 49 | (39.5) |
|  | Unknown | | 71 | (5.7) | 12 | (6.1) | 1 | (7.7) | 6 | (4.8) |
| Size in cm | | | | | | | | | | |
|  | <3 | | 169 | (13.7) | 24 | (12.1) | 2 | (15.4) | 16 | (12.9) |
|  | ≥3 | | 97 | (7.9) | 14 | (7.1) | 1 | (7.7) | 9 | (7.3) |
|  | Unknown | | 969 | (78.5) | 160 | (80.8) | 10 | (76.9) | 99 | (79.8) |
| P_25_: 25^th^ percentile; P_75_: 75^th^ percentile; BMI: body mass index; CIS: carcinoma in situ; DM: diabetes mellitus; TURT: transurethral resection of the bladder tumour | | | | | | | | | | |
| [a] | | 66 patients (4.6%) had a missing value for diagnosis of diabetes mellitus and were included as not diagnosed | | | | | | | | |
| [b] | | Type 1 was defined as diabetes mellitus diagnosis at age ≤30 years, or at age 31-40 years in combination with being not obese; type 2 was defined as diabetes mellitus diagnosis at age >40 years, or at age 31-40 years in combination with being obese | | | | | | | | |
| [c] | | At the time of non-muscle invasive bladder cancer diagnosis | | | | | | | | |
| [d] | | Based on the country of birth of the patients and their parents | | | | | | | | |
| [e] | | Based on the International Standard Classification of Education: low level includes elementary, lower vocational and intermediate general education; intermediate level includes intermediate vocational and higher general education; high level includes higher vocational education and university | | | | | | | | |
| [f] | | Presented for current and former cigarette smokers | | | | | | | | |
| [g] | | Based on the weekly duration of walking, cycling, and sporting during adult life until 2 years before diagnosis; unknown for 25 patients (1.7%) | | | | | | | | |
| [h] | | At the time of filling out the questionnaire | | | | | | | | |
| [i] | | Tumours with WHO 1973 differentiation grade 1 or 2, WHO/ISUP 2004 low grade, or Malmström (Modified Bergkvist) grade 1 or 2a were considered low-grade tumours. Tumours with WHO 1973 differentiation grade 3, WHO/ISUP 2004 high grade, or Malmström (Modified Bergkvist) grade 2b or 3 as high-grade | | | | | | | | |
| [j] | | Based on the European Association of Urology guidelines: low risk includes Ta-stage with low grade, high risk includes Ta-stage with high grade, T1-stage, T2-stage, T3-stage, T4-stage, and CIS | | | | | | | | |
