# Supplementary material for: No clear associations of adult BMI and diabetes mellitus with non-muscle invasive bladder cancer recurrence and progression
Source: PLoS One. 2020 Mar 25;15(3):e0229384. doi: 10.1371/journal.pone.0229384 (PMC7094867; doi:10.1371/journal.pone.0229384)
Supplement: S3 Table — HR: hazard ratio; CI: confidence interval. [a] Defined as a new histologically confirmed tumour in the urinary bladder or prostatic urethra, after ≥1 tumour-negative follow-up cystoscopy result or a radical re-transurethral resection of the primary tumour. [b] The adjustment set consists of age at time of UBC diagnosis, gender, BMI classes, tumour stage, tumour grade, and presence of concomitant CIS. [c] Number of events in the first 5 years after diagnosis of the primary non-muscle invasive urinary bladder cancer. [d] At the time of filling out the questionnaire. (DOCX) [file pone.0229384.s007.docx]

| **S3 Table. Crude and adjusted hazard ratios (HR) with corresponding 95% confidence intervals (CI) for the association of diabetes mellitus with recurrence ^a)^ among non-muscle invasive bladder cancer patients, using different methods for dealing with missing values on diabetes mellitus diagnosis** | | | | | | | | | | | | | |
| --- | --- | --- | --- | --- | --- | --- | --- | --- | --- | --- | --- | --- | --- |
|  | | | *Crude analyses* | | | | |  | *Adjusted analyses* ^b)^ | | | | |
|  | | | Initially at risk | Events ^c)^ |  | HR | (95% CI) |  | Initially at risk | Events ^c)^ |  | HR | (95% CI) |
| Diabetes Mellitus ^d)^ with missing values defined as *not diagnosed* | | | | | | | | | | | | | |
|  | | No | 1,235 | 511 |  | Reference | |  | 1,201 | 496 |  | Reference | |
|  | | Yes | 198 | 95 |  | 1.18 | (0.95-1.46) |  | 191 | 94 |  | 1.22 | (0.98-1.54) |
| Diabetes Mellitus ^d)^ with missing values defined as *not diagnosed*, only if ≥1 question(s) regarding medical history was answered ‘diagnosed’ and none were answered ‘not diagnosed’ | | | | | | | | | | | | | |
|  | | No | 1,212 | 500 |  | Reference | |  | 1,180 | 486 |  | Reference | |
|  | | Yes | 198 | 95 |  | 1.18 | (0.95-1.47) |  | 191 | 94 |  | 1.23 | (0.98-1.54) |
| Diabetes Mellitus ^d)^ with missing values excluded from the analyses | | | | | | | | | | | | | |
|  | | No | 1,169 | 487 |  | Reference | |  | 1,140 | 475 |  | Reference | |
|  | | Yes | 198 | 95 |  | 1.17 | (0.94-1.46) |  | 191 | 94 |  | 1.22 | (0.97-1.53) |
| HR: hazard ratio; CI: confidence interval | | | | | | | | | | | | | |
| [a] | Defined as a new histologically confirmed tumour in the urinary bladder or prostatic urethra, after ≥1 tumour-negative follow-up cystoscopy result or a radical re-transurethral resection of the primary tumour | | | | | | | | | | | | |
| [b] | The adjustment set consists of age at time of UBC diagnosis, gender, BMI classes, tumour stage, tumour grade, and presence of concomitant CIS | | | | | | | | | | | | |
| [c] | Number of events in the first 5 years after diagnosis of the primary non-muscle invasive urinary bladder cancer | | | | | | | | | | | | |
| [d] | At the time of filling out the questionnaire | | | | | | | | | | | | |
