# Supplementary material for: No clear associations of adult BMI and diabetes mellitus with non-muscle invasive bladder cancer recurrence and progression
Source: PLoS One. 2020 Mar 25;15(3):e0229384. doi: 10.1371/journal.pone.0229384 (PMC7094867; doi:10.1371/journal.pone.0229384)
Supplement: S4 Table — HR: hazard ratio; CI: confidence interval; MIBC: muscle invasive bladder cancer. [a] The adjustment set consists of age at time of UBC diagnosis, gender, BMI classes, tumour stage, S5 Table. Crude and adjusted hazard ratios (HR) with corresponding 95% confidence intervals (CI) for the associations of BMI with recurrence, overall progression, and progression to MIBC, among non-muscle invasive bladder cancer patients who received transurethral resection of the bladder tumour (TURT) with one intravesical chemotherapy instillation and adjuvant intravesical immunotherapy. [b] Number of events in the first 5 years after diagnosis of the primary non-muscle invasive urinary bladder cancer. [c] Defined as the first occurrence of stage or grade progression, local or distant metastasis, and cystectomy for therapy-resistant disease. [d] At the time of filling out the questionnaire. [e] Defined as transition to MIBC (stage ≥T2). (DOCX) [file pone.0229384.s008.docx]

| **S4 Table. Crude and adjusted hazard ratios (HR) with corresponding 95% confidence intervals (CI) for the association of diabetes mellitus with progression among non-muscle invasive bladder cancer patients, using different methods for dealing with missing values on diabetes mellitus diagnosis** | | | | | | | | | | | | | |
| --- | --- | --- | --- | --- | --- | --- | --- | --- | --- | --- | --- | --- | --- |
|  | | | *Crude analyses* | | | | |  | *Adjusted analyses* ^a)^ | | | | |
|  | | | Initially at risk | Events ^b)^ |  | HR | (95% CI) |  | Initially at risk | Events ^b)^ |  | HR | (95% CI) |
| **Overall progression ^c)^** | | | | | | | | | | | | | |
| Diabetes Mellitus ^d)^ with missing values defined as *not diagnosed* | | | | | | | | | | | | | |
|  | | No | 1,235 | 143 |  | Reference | |  | 1,201 | 139 |  | Reference | |
|  | | Yes | 198 | 27 |  | 1.16 | (0.77-1.75) |  | 191 | 27 |  | 1.16 | (0.76-1.76) |
| Diabetes Mellitus ^d)^ with missing values defined as *not diagnosed*, only if ≥1 question(s) regarding medical history was answered ‘diagnosed’ and none were answered ‘not diagnosed’ | | | | | | | | | | | | | |
|  | | No | 1,212 | 141 |  | Reference | |  | 1,180 | 138 |  | Reference | |
|  | | Yes | 198 | 27 |  | 1.16 | (0.77-1.75) |  | 191 | 27 |  | 1.15 | (0.75-1.75) |
| Diabetes Mellitus ^d)^ without missing values defined | | | | | | | | | | | | | |
|  | | No | 1,169 | 135 |  | Reference | |  | 1,140 | 133 |  | Reference | |
|  | | Yes | 198 | 27 |  | 1.16 | (0.77-1.75) |  | 191 | 27 |  | 1.14 | (0.74-1.74) |
| **Progression to MIBC ^e)^** | | | | | | | | | | | | | |
| Diabetes Mellitus ^d)^ with missing values defined as *not diagnosed* | | | | | | | | | | | | | |
|  | | No | 1,235 | 36 |  | Reference | |  | 1,201 | 34 |  | Reference | |
|  | | Yes | 198 | 8 |  | 1.37 | (0.64-2.94) |  | 191 | 8 |  | 1.40 | (0.64-3.07) |
| Diabetes Mellitus ^d)^ with missing values defined as *not diagnosed*, only if ≥1 question(s) regarding medical history was answered ‘diagnosed’ and none were answered ‘not diagnosed’ | | | | | | | | | | | | | |
|  | | No | 1,212 | 35 |  | Reference | |  | 1,180 | 33 |  | Reference | |
|  | | Yes | 198 | 8 |  | 1.38 | (0.64-2.98) |  | 191 | 8 |  | 1.42 | (0.65-3.13) |
| Diabetes Mellitus ^d)^ with missing values excluded from the analyses | | | | | | | | | | | | | |
|  | | No | 1,169 | 33 |  | Reference | |  | 1,140 | 32 |  | Reference | |
|  | | Yes | 198 | 8 |  | 1.42 | (0.66-3.07) |  | 191 | 8 |  | 1.39 | (0.63-3.06) |
| HR: hazard ratio; CI: confidence interval; MIBC: muscle invasive bladder cancer | | | | | | | | | | | | | |
| [a] | The adjustment set consists of age at time of UBC diagnosis, gender, BMI classes, tumour stage, tumour grade, and presence of concomitant CIS | | | | | | | | | | | | |
| [b] | Number of events in the first 5 years after diagnosis of the primary non-muscle invasive urinary bladder cancer | | | | | | | | | | | | |
| [c] | Defined as the first occurrence of stage or grade progression, local or distant metastasis, and cystectomy for therapy-resistant disease | | | | | | | | | | | | |
| [d] | At the time of filling out the questionnaire | | | | | | | | | | | | |
| [e] | Defined as transition to MIBC (stage ≥T2) | | | | | | | | | | | | |
