# Supplementary material for: No clear associations of adult BMI and diabetes mellitus with non-muscle invasive bladder cancer recurrence and progression
Source: PLoS One. 2020 Mar 25;15(3):e0229384. doi: 10.1371/journal.pone.0229384 (PMC7094867; doi:10.1371/journal.pone.0229384)
Supplement: S5 Table — HR: hazard ratio; CI: confidence interval; BMI: body mass index. [a] Defined as a new histologically confirmed tumour in the urinary bladder or prostatic urethra, after ≥1 tumour-negative follow-up cystoscopy result or a radical re-transurethral resection of the primary tumour. [b] Defined as the first occurrence of stage or grade progression, local or distant metastasis, and cystectomy for therapy-resistant disease [c] Defined as transition to MIBC (stage ≥T2). [d] The adjustment set consists of age at time of UBC diagnosis, gender, highest completed level of education, weekly duration of physical activity, history of urinary bladder cancer among first degree relatives, tumour stage, tumour grade, and presence of concomitant CIS. [e] Number of incident events in the first 5 years after diagnosis of the primary non-muscle invasive urinary bladder cancer. [f] Based on average BMI during adult life. (DOCX) [file pone.0229384.s009.docx]

| **S5 Table. Crude and adjusted hazard ratios (HR) with corresponding 95% confidence intervals (CI) for the associations of BMI with recurrence, overall progression, and progression to MIBC, among non-muscle invasive bladder cancer patients who received transurethral resection of the bladder tumour (TURT) with one intravesical chemotherapy instillation and adjuvant intravesical immunotherapy** | | | | | | | | | | | | | |
| --- | --- | --- | --- | --- | --- | --- | --- | --- | --- | --- | --- | --- | --- |
|  | | | *Crude analyses* | | | | |  | *Adjusted analyses* ^d)^ | | | | |
|  | | | Number at risk | Events ^e)^ |  | HR | (95% CI) |  | Number at risk | Events ^e)^ |  | HR | (95% CI) |
| **Recurrence ^a)^** | | | | | | | | | | | | | |
| BMI classes in kg/m^2 f)^ | | | | | | | | | | | | | |
|  | ≥18.5 and <25.0 | | 130 | 50 |  | Reference | |  | 126 | 49 |  | Reference | |
|  | ≥25.0 and <30.0 | | 119 | 44 |  | 0.97 | (0.64-1.45) |  | 116 | 44 |  | 1.10 | (0.72-1.69) |
|  | ≥30.0 | | 26 | 10 |  | 0.92 | (0.47-1.81) |  | 26 | 10 |  | 1.02 | (0.50-2.07) |
| **Overall progression ^b)^** | | | | | | | | | | | | | |
| BMI classes in kg/m^2 f)^ | | | | | | | | | | | | | |
|  | ≥18.5 and <25.0 | | 130 | 31 |  | Reference |  |  | 126 | 30 |  | Reference |  |
|  | ≥25.0 and <30.0 | | 119 | 26 |  | 0.89 | (0.50-1.46) |  | 116 | 26 |  | 1.04 | (0.60-1.80) |
|  | ≥30.0 | | 26 | 6 |  | 0.89 | (0.37-2.13) |  | 26 | 6 |  | 1.09 | (0.43-2.77) |
| **Progression to MIBC ^c)^** | | | | | | | | | | | | | |
| BMI classes in kg/m^2 f)^ | | | | | | | | | | | | | |
|  | ≥18.5 and <25.0 | | 130 | 9 |  | Reference |  |  | 126 | 8 |  | Reference |  |
|  | ≥25.0 and <30.0 | | 119 | 7 |  | 0.84 | (0.31-2.25) |  | 116 | 7 |  | 1.45 | (0.50-4.22) |
|  | ≥30.0 | | 26 | 2 |  | 1.10 | (0.24-5.11) |  | 26 | 2 |  | 2.50 | (0.47-13.28) |
| HR: hazard ratio; CI: confidence interval; BMI: body mass index | | | | | | | | | | | | | |
| [a] | | Defined as a new histologically confirmed tumour in the urinary bladder or prostatic urethra, after ≥1 tumour-negative follow-up cystoscopy result or a radical re-transurethral resection of the primary tumour | | | | | | | | | | | |
| [b] | | Defined as the first occurrence of stage or grade progression, local or distant metastasis, and cystectomy for therapy-resistant disease | | | | | | | | | | | |
| [c] | | Defined as transition to MIBC (stage ≥T2) | | | | | | | | | | | |
| [d] | | The adjustment set consists of age at time of UBC diagnosis, gender, highest completed level of education, weekly duration of physical activity, history of urinary bladder cancer among first degree relatives, tumour stage, tumour grade, and presence of concomitant CIS | | | | | | | | | | | |
| [e] | | Number of incident events in the first 5 years after diagnosis of the primary non-muscle invasive urinary bladder cancer | | | | | | | | | | | |
| [f] | | Based on average BMI during adult life | | | | | | | | | | | |
